# Abstract of the Proceedings of the Buffalo Medical Association

**Published:** 1858-11

**Authors:** Austin Flint


### ART. IV.—*Abstract of Proceedings of the Buffalo Medical Association.*

TUESDAY EVENING, Oct. 5, 1858.

The Association met.

Present—The President, Dr. Wyckoff, in the chair; Drs. Gould, White, Rochester, Flint, Miner, Rogers, Butler, Jansen, Gay, Congar, Newman, Wilcox, Treat, Eastman, and Flint, Jr.

The minutes of the last meeting were read and approved.

Prof. ROCHESTER exhibited the case of dislocation of the sternal extremity of the clavicle, which he reported at the last meeting. Measurement showed no difference in the distances of the acromium processes from the sternum, but a prominence and mobility of the articulation indicated the form of dislocation. The patient had a pretty good use of the arm. There was a partial separation of the extremities of the lower ribs from their cartilages.

Dr. GOULD then reported the following cases of *Hæmorrhoids treated by the Application of Nitric Acid.* In the absence of Dr. Gould, these cases were read by the secretary.

CASE I. W. W., aged 28 years, German, shoemaker by occupation. He has suffered three years from hæmorrhoidal discharges, which occurred every time he had a passage from his bowels. His sufferings on these occasions were described as almost unendurable, he being obliged to remain at stool a long time, and the exhaustion from pain and loss of blood causing even such a degree of prostration that he would be unable to work for hours after. He was low spirited, nervous and ænemic. He applied to me for treatment on

May 7th, 1857. On examining the protruding parts, I found them to consist of several hæmorrhoidal tumors, from the size of a pea to that of a pigeon's egg. The surface of the protruding mass was of a dark purple or livid color, on the spongy surface of which there appeared a continued sanguinous discharge. Considering it a suitable case for the application of nitric acid, I ordered a dose of ol. ricini to be taken at bedtime, and repeated in the morning.

May 8th, 2, P. M. The oil had operated well.

Gave an enema of warm water, and directed him to sit over the vessel and force down the tumors as much as possible; he was then placed across the bed, face downwards, an assistant separating the nates, and I applied, with a brush, strong nitric acid to the entire surface. After a quarter of an hour a piece of oiled lint was applied, and the parts gently returned. The patient was then directed to remain quiet in bed, and pulv. Doveri, grs. x, to be taken and repeated at bed time.

May 9th. Patient comfortable and in good spirits.

Ordered the Dover's powder to be repeated every night at bedtime.

May 16th. Found, on examination, that the tumors were reduced in size one-half, and hardened, as if blood were densely coagolated within them. They were brought down as before, and the acid again applied. The after treatment the same as after the first application.

May 17th. Complains of itching and uneasiness in rectum.

Ordered parts bathed with cold water.

May 18th. Uneasiness in rectum continues.

Ordered the cold bathing continued, and an unguent, composed as follows, applied to the parts:

℞ Ac. Pl., ʒj;

Lard, ℥j;

Nitric acid, gtts. viii. M.

Apply morn, noon and night.

May 20th. The uneasiness and itching ceased; the tumors very much reduced in size, and the parts assuming a natural and healthy appearance.

Ordered an enema of cold water, to move the bowels, as he had had no evacuation since the last application.

May 23d. Has had two evacuations without using the injections. Very little blood has been lost since the first application.

Ordered sulphur to be taken as a laxative, every morning for a week or two.

Mr. W. has enjoyed good health to the present time, Oct. 4th, 1858, and has had no return of the disease.

The pain attending the applications were represented by the patient as far less than that suffered at each former dejection.

Case II. Mrs. T., aged 46 years, English; had suffered with hæmorrhoids for fourteen years. First noticed them protruding after confinement. Had been frequently returned, but would not remain within the sphincter.

August 26th. On examination, found three large tumors, which collectively formed a mass as large as a common sized tomato, and presenting much the same appearance completely surrounding the anus. From the centre there was a constant sanguinous oozing. The surface very irritable. Considering it a suitable case for the nitric acid treatment, I decided to apply it, which was done in the same manner as in case first, assisted by Dr. Austin Flint, Jr.

30th. Tumors reduced to one-half their former size.

Sept. 5th. The tumors very much diminished. Patient feels well. Assisted by Dr. Flint, Jr., I applied the acid to the remaining portion of the tumors. The pain was slight, the patient acknowledging that she was more frightened than hurt.

Has had no trouble since the last application. Thinks she is permanently cured.

Case III. Mrs. McG., aged 25 years, Irish; has been troubled with hæmorrhoids fourteen months. Was confined Sept. 2d, 1858. On examination, I found a cluster of hæmorrhoidal tumors as large as a common tomato. Some were red, others purple, and very irritable.

Sept. 5th. I applied nitric acid to the whole surface of the tumors. Found it impossible to return. Applied a piece of oiled lint and left them out.

Sept. 6th. Patient very comfortable. Pain lasted about half an hour. Had a passage from bowels without pain.

11th. The tumors are reduced to one-third their former size, and not painful. Patient is up and doing her housework.

15th. Bowels regular; defecation easy. Applied the acid to what remained of the tumors.

Oct. 4th. Up to this time no return of the disease.

It will be noticed that the remedy was applied in this case only three days after confinement.

Dr. Flint, Jr., remarked that he was present at the application of the nitric acid in the last two cases reported by Dr. Gould, and wished to add his testimony to its efficacy. In both the cases the tumors were both of the internal and external variety, and in neither of them could they be restored within the sphincter after the application. The action of the acid was very vigorous and appeared to shrivel them almost immediately; the pain of the application being comparatively slight, and, as described by one of the patients, no more than she had frequently suffered in having an operation from the bowels. He was also present at the second applications which were made in both cases, seven or eight days after the first; at that time, the tumors had diminished in size at least one-half. The second application had the effect of removing them entirely, and, in the course of from one to two weeks, restoring the anus to its natural appearance.

An account of this mode of practice appeared some time since in the journals, but Dr. Flint was not aware that it had ever been practiced to any extent in this vicinity. He regarded it as one of the most effectual and painless operations for hæmorrhoids, infinitely preferable, on the score of suffering to the patient, to the operation by the ligature.

Dr. Congar had seen a case where he thought this application would not have answered a very good purpose, when the hæmorrhoids were external and could not be returned. He applied a ligature, and they came off in twelve or fourteen days.

Dr. Flint, Jr. replied that in the cases reported by Dr. Gould, the tumors were both external and internal, and that the nitric acid application seemed to produce as good effect in the one as the other. Of course the treatment was not supposed to be applicable in *all* cases, but it seemed to him that it would be effectual in most of them.

Prof. Rochester said that he had employed an unguent of the perchloride of iron mixed with lead, in less severe cases, with good effect.

Prof. Rochester then reported the following case:

*A Case of Pyogenic Croup, terminating in recovery.* By Thomas F. Rochester, M. D.

Simon A., aged four years, of Jewish parentage, and of delicate and strumous habit, had suffered from a slight cold for eight days; it had not confined him to the house, and had apparently yielded to domestic treatment. On Thursday, Sept. 16th, he ate freely of apples, and in consequence was repeatedly purged during the ensuing night. It was also noticed that he was extremely restless, that he coughed a good deal, and that he breathed laboriously. I was summoned to see him on the 17th, at 8, A. M. He was tossing upon the bed, his countenance was cadaveric, the whole surface cold and pale; respiration about 80; pulse 140, and very feeble; voice weak but distinctly articulate, and not in the least hoarse and husky; cough slight, infrequent and soft; respiratory murmur very feeble. The idea of a foreign body in some portion of the air passages immediately suggested itself; its presence, however, could not be determined. Leaving the diagnosis unsettled, external warmth was directed, and Tr. opii, camp. f℥j, was immediately administered, to be repeated in an hour if the purging continued. Strong hot brandy sling was also ordered to be given freely, and half a grain of calomel rubbed up with sugar to be placed upon the tongue every half hour.

11, A. M. Surface warm, good reäction, but the respiration, if possible, more rapid and heaving; pulse increased in force and undiminished in frequency. There has been no further purging. The patient's strength permitting, a careful examination was now conducted. There was no evidence of colic, of peritonitis, of pleuritis, of collapse of lung, or of pericarditis. Inspection of the fauces revealed no false membrane or even unusual injection. Auscultation over the trachea, gave a slight mucous râle, somewhat increased in intensity, over the bifurcation. The respiratory murmur was very feeble and difficult to detect. The expiratory sounds were prolonged and more distinct than the inspiratory. Percussion over the thorax gave everywhere resonance, slight, however, and much less than in health, (as was determined after recovery.) At every effort at inspiration, the lower portion of the trachea was strongly retracted and then forcibly protruded. This movement was not extended to the diaphragm, as is usually seen in the advanced stages of membranous croup. It was evident that the *obstruction*, whatever its nature, was in the lower portion of the trachea, and as reäction had been effected by stimulants, it was determined to attempt to dislodge it by an emetic. With this view the following prescription was made:

℞ Syr. ipecac, ℥ij;
Zinc. Sulph. ℈j. M.

One tablespoonful every twenty minutes until emesis is produced.

I remained and administered the first dose. In fifteen minutes it acted freely. Among the ejecta were several masses of muco-purulent appearance, but no undigested food, and no fragment of apple or other foreign substance. Decided relief was apparent. It being evident that the muco-purulent exudation was the cause of the difficult breathing, it was judged advisable to prevent if possible the further extension of the disease by topical measures, a solution of nitrate of silver, thirty grains to the ounce, was accordingly applied freely, by a sponge-probang, to the pharynx and larynx. A large hot fomentation was directed to be kept to the chest, principally to insure a warm moist atmosphere for inhalation.

Calomel to be continued every hour and the hot brandy sling to be taken occasionally.

3, P. M. Being called in haste, I found the child breathing with the utmost difficulty; condition otherwise unchanged; the voice remaining clear, but feeble. I immediately repeated the emetic. It afforded prompt relief, bringing away a large amount of the same exudation as the first one. A portion of this had meanwhile been examined, microscopically, by Dr. John Boardman, and was found to consist of mucus, altered blood discs, and pus globules.

The nitrate of silver was reäpplied, and the treatment continued, with the addition of one grain of sulphate of quinine in solution, every hour.

7, P. M. Child sleeping for the first time in eighteen hours. Pulse 120; respiration 40.

Directed beef essence, calomel and quinine to be alternated every hour.

9½, P. M. Dr. Newman in consultation. Child sleeping; countenance pale; respiration 45, and jerking. On awakening he spoke and cried clearly. Respiratory murmur very feeble; pulse 120.

Treatment continued. The emetic to be repeated if the breathing should become more embarrassed. Tr. opii camp. f℥j, if the patient should be very restless.

Saturday, Sept. 18th, 8, A. M. Improvement very decided. During the night, it was thought advisable to repeat the emetic. It brought away considerable of the muco-purulent secretion, after which the child slept quietly most of the night. Early in the morning it had an alvine evacuation, characterized by the constitutional effect of the mercury. Pulse 100; respiration 30; skin warm and moist; respiratory murmur distinct, accompanied by slight mucous râles; cough slight, soft and infrequent; voice clear and strong.

Treatment, quiniæ sulph., gr. j, every three hours, and nourishing diet.

8, P. M. Still improving.

Sunday, Sept. 19th. Patient discharged convalescent.

There are several points of especial and unusual interest in the above case. First. The location and limited extent of the disease. Second. The sudden accession of alarming symptoms unattended with loss or hoarseness of voice or cough. And, lastly, the rapidity and completeness of the convalescence.

With reference to the treatment, it is assumed that the emetics were the active curative agents, and it is believed that they alone were competent to the removal of the obstruction; it is not likely that it could have been reached, or if reached, removed by the probang. Topical medication with solution of nitrate of silver, was employed as a protective measure, and as the larynx was not invaded, its exemption may, perhaps, be attributed to that precaution. The use of calomel was possibly unnecessary, it is of course out of the question to affirm with certainty that the exudation was arrested by its action, but it was not considered safe to omit it, and it surely neither produced nor was followed by any ill effects. It is also believed, that while the ventilation of the room was secured, keeping a warm and moist air immediately about the patient was of great efficacy.

It is, perhaps, note-worthy, that in February, 1855, the writer lost, in the same house, a child of about the same age, and also of Jewish parentage, with membranous croup.

Prof. FLINT called to mind a case of death from accumulation in the air passages, where the disease was not membranous croup. This was, to him, a very instructive case, as it was accompanied by all the symptoms of true croup. In this case he thought that active emesis might have removed the obstruction and saved the life of the patient.

Dr. NICHELL reported the following case of swallowing a foreign body:

Margaretha Dangbon, æt. 6 years, swallowed an irregular piece of colored glass about an inch in diameter, and two or three lines in thickness, on the 8th of September, 1858, at 10, A. M. The child did not suffer very much from pain, but having an uneasy feeling in the stomach, the mother gave her a cupful of molasses shortly after. Dr. Nichell soon saw her, and thought it unnecessary to do anything more. The piece of glass was passed *per rectum* the next morning, at half past seven, without having done any harm to the alimentary canal.

The specimen was presented to the association.

Prof. ROCHESTER saw a case, with Dr. Nichell, of remarkable malformation. It was a child three days old, who had never urinated and was unable to do so. On examination it appeared that the membrane of the meatus urinarius was continuous with the skin of the prepuce, and on slitting up the prepuce and finding the urethra, the difficulty was removed.

Prof. FLINT reported another case of asthma which was produced by the emanations from a feather bed. It was a child 5 years of age who had always suffered from asthmatic breathing. The father and mother were both subject to it in some degree. They had always been accustomed to sleeping on feather beds, and on making a change in this regard, according to the suggestion of Prof. Flint, there was no return of the difficulty. The change in the case of the mother was not so marked as in the other case, as the change of the bed, induced a cold from which she had not yet recovered.

Dr. Miner mentioned a similar case; this patient, in addition, could never breathe well where there was any new-mown hay; the odor from a horse, also, was sufficient to bring on the difficulty, so much so, indeed, that he preferred walking a long distance to riding after a horse. He had a son who suffered from the same idiosyncracy.

Dr. Butler then reported the following case of *Rupture of the Uterus:*

The patient was a German woman, of good constitution, aged 24, delivered of her third child, who died thirty-three hours after delivery.

The patient was attended by a German midwife, but on the arm presenting, she sent for a physician who turned and delivered.

The midwife makes the following statement: "Saw the patient Friday, between 7 and 8, P. M.; had slow pains and the uterus was dilated to the size of a twenty-five cent piece; was up and about; at that time could not tell whether it was a knee or an arm presentation; membranes had not broken; they broke three or four hours after, when the right arm came down; womb fully dilated before the waters came away; pains before had been rather inefficient, but afterwards were very strong, lasting about three minutes, then an intermission of six minutes—between pains very quiet. Before the waters broke, gave her a powder of ergot,—she vomited it up. Examined and found the right shoulder to the left anterior of pelvis, could only reach the neck of child; then sent for a doctor, he came between 1 and 2; patient did not flow before the doctor came; had a chill before the waters broke; *only vomited once;* did not observe any change in her countenance. When the doctor came he proceeded to turn, placing the woman on her arms and knees, and in ten minutes the child was born; patient did not complain very much; when we put the woman in bed discovered bleeding, but no more than ordinary; found the placenta in vagina, and took it away; remained an hour after delivery; patient did not vomit; child born dead; did not give any medicine or apply bandage after delivery; the womb was contracted before I left; there was more tenderness than usual; left the woman as comfortable as women are generally after confinement; she got exhausted after delivery,—short breath, perspiration, &c.

"When I saw the womb bulging up, I got quite uneasy, but it contracted down after awhile."

The attending physician says: "The right arm presented; passed the left hand into the womb; the head of the child lay in the right iliac fossæ, and the right shoulder was also pressed down in the same place; the face was anteriorly. The whole operation lasted about ten minutes, and was accomplished remarkably easily,—never made version so easily. The woman complained very much during version, and afterwards referred the pain to the *right side;* wanted to be laid on right side after operation. The mouth of the womb quite soft and dilatable; did not make any resistance; did not notice anything unusual only that it was done so easily; did not mistrust anything wrong; the womb was like a bag, and the parts so relaxed a person would have thought it might have come away itself, only there were no uterine contractions; child large—full term; it had a depression on the left side of the head; had been hæmorrhage when I got there; patient did not vomit when there; woman had a constant pain in abdomen."

A woman who was present says: "The doctor ordered the patient to lie on her hands and knees, because he wanted to use force. When the child was born to the arms, the patient was complaining very much; at this time the doctor had his hand within the patient; after the child was turned, force was used in pulling. The woman did not complain of anything extraordinary except when the *arms were born;* did not scream out suddenly at any time; the woman only complained of pain in the back before delivery, but afterwards of pain in the abdomen; in fact did not complain of pain in any particular place until after delivery, and then referred to the right side."

[It is proper for me to refer to the apparent discrepancies of the statements of the above parties. All the facts were obtained through an interpreter; translation may be at fault in some instances, from misconception of the shades of meaning, or of the questions. It would be natural for both midwife and doctor to make the case as favorable as possible to their own side, for fear of unpleasant consequences. All these things must be taken into account in making up a judgment in the case.]

*Post-mortem* by Dr. B., twenty-seven hours after death. Present, Drs Winne, Loomis and Nott. Abdomen only examined.

*External appearances.* Face pale; eyes dull and glazed; bloody froth issuing from mouth; abdomen very much bloated; some blood flowing from vagina; remains of distended veins on abdomen; cellular tissue infiltrated with gas; labia majora very much congested; dark rim of venous congestion at Poupart's ligament; mucous membrane of right labia majora torn at least an inch.

On opening the pelvic cavity, found the lower part filled with bloody serum; no marked peritoneal inflammation; lower part of external and internal oblique muscles of right side much gorged with blood; peritoneal coat of right ovarium deeply discolored—even black; bladder empty.

Found rupture of the uterus, extending from and involving os and neck, to right round ligament, through entire lower third of uterus—at least five inches long. There was no apparent thinning of the walls; edges, perhaps, a little softened, which might be due to remaining in the mass of blood, mucus, &c. The uterus was quite offensive and the bowels filled with offensive flatus.

It was the opinion of the medical men present, that rupture occurred at the time of turning, for the following, among other reasons:

Blood did not flow from vagina before turning; the woman did not complain of pain in right side before, but in the back or elsewhere; the child would have gone into the abdomen; turning is one of the causes of rupture of the uterus. There was no marked disproportion between the size of the head and the capacity of the pelvis; no apparent thinning of uterine walls. It was also the opinion that no blame could be charged to the medical attendant, as the accident might occur to any person.

The specimen was then presented to the association, and on motion, Dr. Butler was authorized to prepare it in a proper form for preservation, at the expense of the association.

Dr. Flint, Jr. moved the consideration of the resolutions which were presented at the last meeting, which motion was seconded, *i. e.*:

*Resolved*, That the Association fully concurs in the sentiments expressed in the editorial article entitled "*Criminal Abortions*," in the Sept. number of the Buffalo Medical Journal, relative to its frequency and criminality.

*Resolved*, That a committee of three be appointed from this Association, to confer with the county and city authorities, as to the laws now existing, if properly enforced, and whether further legislation is necessary for the abolishment of this great and growing evil.

*Resolved*, That this committee invite the coöperation of the Medical Societies and Associations in this State, in any measure which may be deemed necessary and expedient to lessen these horrible offences against the morality of the community.

The above resolutions were then freely and feelingly discussed by nearly all the members present, and on the question being put, were adopted unanimously.

The chair then appointed Drs. White, Wilcox and Flint, Jr., such committee.

The Association then adjourned.

AUSTIN FLINT, Jr., M. D.,
Secretary.

